# Biological Therapies in the Treatment of Cancer—Update and New Directions

**DOI:** 10.3390/ijms222111694

**Published:** 2021-10-28

**Authors:** Monika A. Papież, Wirginia Krzyściak

**Affiliations:** 1 Department of Cytobiology, Faculty of Pharmacy, Jagiellonian University Medical College, 30-688 Kraków, Poland; 2 Department of Medical Diagnostic, Faculty of Pharmacy, Jagiellonian University Medical College, 30-688 Kraków, Poland

**Keywords:** biological therapy, cancer, recombinant antibodies, CAR T cells, oncolytic viruses, cancer vaccines, cancer microenvironment

## Abstract

Biological therapies have changed the face of oncology by targeting cancerous cells while reducing the effect on normal tissue. This publication focuses mainly on new therapies that have contributed to the advances in treatment of certain malignancies. Immunotherapy, which has repeatedly proven to be a breakthrough therapy in melanoma, as well as B-ALL therapy with CAR T cells, are of great merit in this progress. These therapies are currently being developed by modifying bispecific antibodies and CAR T cells to improve their efficiency and bioavailability. Work on improving the therapy with oncolytic viruses is also progressing, and efforts are being made to improve the immunogenicity and stability of cancer vaccines. Combining various biological therapies, immunotherapy with oncolytic viruses or cancer vaccines is gaining importance in cancer therapy. New therapeutic targets are intensively sought among neoantigens, which are not immunocompromised, or antigens associated with tumor stroma cells. An example is fibroblast activation protein α (FAPα), the overexpression of which is observed in the case of tumor progression. Universal therapeutic targets are also sought, such as the neurotrophic receptor tyrosine kinase (NTRK) gene fusion, a key genetic driver present in many types of cancer. This review also raises the problem of the tumor microenvironment. Stromal cells can protect tumor cells from chemotherapy and contribute to relapse and progression. This publication also addresses the problem of cancer stem cells resistance to treatment and presents attempts to avoid this phenomenon. This review focuses on the most important strategies used to improve the selectivity of biological therapies.

## 1. Introduction

Cancer is one of the leading causes of death in the world, generates enormous costs and is a major burden on humanity. According to the GLOBOCAN online database report from 2020, it is forecast that the annual number of cancer cases in the world will increase from 19.3 million in 2020 to 28.4 million in 2025 (an increase of 47% compared to 2020) [1]. Oncologists emphasize that classical chemotherapy is already reaching the limits of its effectiveness, therefore, other methods are needed that would enable progress in the treatment of many types of cancer [2]. This problem particularly affects older patients, who most often suffer from these diseases and, at the same time, due to their age and other loads, tolerate chemotherapy much less well than young patients. The hope is in biological therapies that can reduce side effects by acting more selectively on cancer cells.

Biological cancer therapy involves treatment with natural molecules made by the body or made in a laboratory. These therapies either help the immune system fight the cancer or attack the cancer directly. These include treatment with monoclonal antibodies, adoptive cell transfer, gene therapy, treatment with cytokines, cancer vaccines, oncolytic viruses, immunoconjugates and the use of targeted therapy. Biological therapies used in cancer treatment are currently booming and are targeted therapies that fit perfectly into the emerging trend of precision oncology, which uses the results obtained by next-generation sequencing (NGS) methods to detect new, rare mutations in cancer cells in order to tailor treatment to a specific patient [3]. In the biological therapy of cancer, molecules that target genetic aberrations in oncogenes and tumor suppressor genes leading to tumor development are essential. Classic examples of such molecules are: imatinib, a BCR-ABL tyrosine kinase inhibitor used in chronic myeloid leukemia; vemurafenib, a BRAF seronine/threonine kinase inhibitor for the treatment of melanoma; or osimertinib, approved by the FDA and the EC in 2017 for the treatment of non-small cell lung cancer in the presence of the EGFR T790M mutation [4,5,6,7].

Monoclonal antibodies play a huge role in cancer therapy. The first data on the effectiveness of the use of murine monoclonal antibodies against antigens overexpressed in neoplastic cells came from studies in laboratory animals [8]. The problem with the use of these antibodies was that they were multi-species and thus not very effective because they did not work well with the components of the human immune system, and in addition, they were immunogenic and were neutralized by the human immune system [9]. Only the development of the methods of obtaining recombinant antibodies opened the way to therapy, which has already contributed to success in oncology many times, see review [10]. These recombinant antibodies were created by combining the variable part of the murine antibody with the constant part of the human [11]. Chimeric antibodies with reduced immunogenicity were obtained, which, thanks to the human Fc fragment, could cooperate with cells of the human immune system and with complement components. Then, by further reducing the proportion of the murine variable part, humanized antibodies were obtained that are 85–90% human [9,12].

It is important to look at cancer in a multidirectional way, in the development of which the microenvironment also participates, with numerous modulating factors affecting the adhesion, migration, proliferation and drug resistance of cancer cells. The combination of strategies targeting tumor cells and normal tumor-associated cells may have greater therapeutic effects. This study concerns not only the biological therapies already developed but also informs about the directions of research based on the increasingly better understood cancer biology, which may lead to the development of a much more effective anti-cancer treatment.

## 2. A Strategy for Treating Cancer by Unblocking Effector Lymphocytes as a Type of Immunotherapy

In the organism, the action of the activating mechanism is controlled by an opposing inhibitory mechanism that prevents over-activation. In the immune system, such brakes are the control points of the immune response. These include receptors on defense cells, which under physiological conditions prevent autoaggression and tissue damage by overactive T lymphocytes during the immune response [13]. Neoplastic cells use this mechanism to switch off effector T lymphocytes by exposing on their surface ligands for receptors belonging to the immune checkpoints, such as PD-1 [14]. Although lymphocytes infiltrate the tumor, they are not able to destroy cancer cells. Another important receptor taking part in the negative regulation of T lymphocytes is CTLA-4 expressed on dendritic cells [15] (Table 1). After the discovery of this mechanism and the production of antibodies neutralizing these receptors, the effector T lymphocytes in the tumor microenvironment were unblocked and allowed to cause the lysis of neoplastic cells [16]. This method has become a real revolution in the treatment of certain types of cancer.

Melanoma is a model cancer in which such therapy has brought a breakthrough in treatment in the last decade. Compared to dacarbazine, which was previously used as one of the primary cytostatic drugs for the treatment of melanoma, the annual overall survival rate increased approximately twofold after ipilimumab anti-CTLA-4 therapy compared to dacarbazine, threefold after treatment with nivolumab or pembrolizumab anti-PD-1 therapy and fourfold after combining the two therapies [17]. Following the introduction of therapies with these antibodies, it was thought that they would only be effective against immunogenic tumors such as melanoma. In the course of the research, however, it was found that they also have positive therapeutic effects in the case of non-immunogenic neoplasms, which include, for example, lung cancer [18]. Applications of this therapy have also been approved for cancers of the kidneys, head and neck, Hodgkin’s lymphoma, urinary tract, colon, rectal, hepatocellular carcinoma of the breast, cervix, lung, skin and stomach [19].

It should be added that the possibilities of therapy based on the suppression of immune checkpoints have not been exhausted yet, as many other molecules inhibiting the function of cytotoxic lymphocytes present within the immune synapse are not known. Inhibitors are under development for lymphocyte activation gene 3 (LAG-3; CD223), which compete for TCR binding with MHC II molecules, thereby inhibiting the proliferation and differentiation of T lymphocytes. Potential therapeutic targets are also another component of immune synapse, e.g., T cell immunoglobulin and mucin-3 (TIM-3), band T-cell lymphocyte attenuator (BTLA), V-domain Ig suppressor of T-cell activation (VISTA) and TIGIT [20].

## 3. Recombinant Antibodies in the Treatment of Cancer

**Figure 1:**
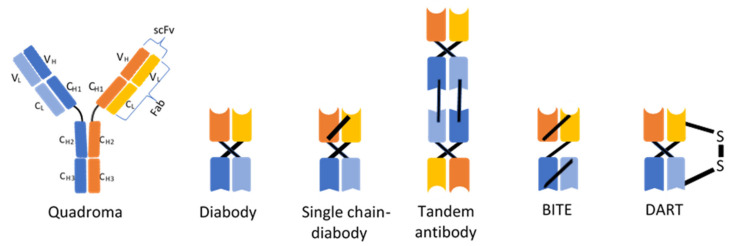
Selected recombinant antibodies developed for anti-cancer therapies. BITE—bispecific T-cell engager, DART—Dual affinity retargeting.

The first generation of bispecific antibodies is the product of the quadrome, a cell line generated by the fusion of two hybridomas, see review [21]. The resulting molecules are a combination of the heavy and light chains of the two antibodies (Figure 1). When hybridomas from two different species are fused, light and heavy chains of the same species are preferentially bound [22]. Such cell lines hardly produce antibodies with chain mismatches. On the other hand, the fusion of two single-species hybridomas leads to the production of mismatched antibodies that cannot fulfill the expected functions [21]. In such a situation, only 1 molecule out of 10 is a hybrid antibody. These antibodies are extracted with protein A by chromatography [23]. The antibodies produced by the quadrome are bispecific and trifunctional [23,24,25]. This means that one variable fragment of an antibody recognizes one antigen, most often a CD3 molecule on the surface of a cytotoxic T lymphocyte, and the other fragment binds another antigen, which is a tumor cell marker. Therefore, these antibodies are used to attract a cytotoxic lymphocyte to a neoplastic cell, which enables the activation of this defense cell and neoplastic lysis [26]. This results in a more effective stimulation of the host’s immune system to destroy cancer cells. This is a completely unique property of antibodies. Due to the fact that these antibodies have the classic structure of the IgG molecule and contain the constant Fc fragment, they can bind to the receptor for this fragment, which is present on granulocytes, macrophages and dendritic cells [26]. The third function of these antibodies is therefore to bind and activate an additional cell. As a result, the T-lymphocyte is co-activated by contact with the neoplastic cell and with the participation of an accessory cell that produces stimulating cytokines. Finally, an activated accessory cell can phagocyte a neoplastic cell [27]. This property of bispecific antibodies significantly increases their effectiveness. By creating bispecific antibodies, molecules with unique properties were obtained which allow them to obtain an effect unattainable for other known therapies. Among the trifunctional antibodies for therapy, for example, catumaxomab specific for CD3 and EpCAM, which is used to reduce peritoneal exudate in exudative ovarian cancer, has been introduced [28,29]. Similarly, ertumaxomab (Rexomun^®^, Fresenius Biotech GmbH, Bad Homburg, Germany) targeting CD3 and the HER-2-neu oncogene significantly increased the number of lymphocytes targeting tumor cells in peritoneal carcinomatosis [30]. Ertumaxomab has also proven to be effective in Phase 1 and 2 clinical trials in HER-2 positive breast cancer, also in the case of low expression of this receptor [31,32]. The administration of mosunetuzumab, the bispecific monoclonal IgG1 antibody against CD3 and CD20, has been shown to result in a sustained response in patients with B cells non-Hodgkin lymphoma (NHL). Mosunetuzumab is effective in patients with poor prognosis and relapsed and refractory NHL to CAR T cells therapy [9].

However, the first generation of bispecific antibodies have drawbacks that make them ineffective in treating solid tumors. Problems with these antibodies are discussed extensively in the review by Chames et al. (2009) [33]. First of all, the problem is the size of the antibody molecule with the structure of the classic IgG molecule [34,35,36,37]. It is known that the size of the molecule is decisive for diffusion. Therefore, these particles have little penetration into the tissue of tumors with tortuous heterogeneous vascularization and high interstitial fluid pressure [34]. Appropriate affinity for the antigen is also essential for the good penetration of the tumor tissue by antibodies. Too high affinity causes the so-called binding site barrier effect [35,36,38]. The antibody binds with great force to the first antigen it encounters at the periphery of the tumor, close to the blood vessels, and does not penetrate further. On the other hand, moderate affinity antibodies dissociate easily from the first antigen encountered and penetrate deep into the tissue, leading to an even distribution in the tumor. There is also a problem with the binding of antibodies to the activating receptor for the Fc fragment, as approximately 80% of the population have a low affinity variant of this essential receptor for antibody-dependent cell-mediated cytotoxicity (ADCC) [39]. Consequently, these individuals will not fully benefit from the effects of the therapeutic antibodies. Moreover, IgG1 are glycosylated in the CH2 domain of the Fc region, which further modulates the Fc affinity for FcγRIIIa, thereby modifying the in vivo efficacy of the antibodies [40]. Another problem is the high concentration of the patient’s natural antibodies. Therapeutic antibodies must compete with the high concentration of the patient’s IgG in binding to FcγRIIIa, hence the necessity to use high concentrations of these antibodies [41]. The effectiveness of therapeutic antibodies may be adversely affected by their affinity for inhibitory Fc receptors, such as FcγRIIb, expressed in B lymphocytes, macrophages, dendritic cells and neutrophils [9]. One of the main strategies used to improve the effectiveness of antibodies in the fight against cancer is increasing their affinity for activating receptors and reducing their interaction with the inhibitory FcγRIIb receptor [42].

A great achievement was the development of second-generation bispecific antibodies, which are antibody fragments. Single-chain scFv variable fragments containing variable domains of VH–VL heavy and light chains joined by a flexible linker (Figure 1) were used. Based on this technology, antibody fragments of various sizes are created, which retain the binding activity of IgG antibodies [43]. The problem with the size of the molecule was thus solved by removing the Fc fragment of antibody, and the binding affinity and stability were optimized [43].

Examples of such fragments are diabodies, i.e., small dimeric, bivalent or bispecific antibody fragments (Figure 1). Two single-chain VH–VL fragments were cross-linked, and the linker length between VH–VL was reduced from 15–20 to 5 amino acids [44,45]. In the case of bivalent diabodies, being a combination of identical VH–VL chains (homodimers), an increase in affinity was achieved due to two antigen binding sites [45]. A variant of these recombinant antibodies are Bispecific Diabodies (BsDbs), which contain two VH and VL domains derived from two different antibodies, specific for different antigens [46] (Figure 1). The compact structure of such a molecule ensures rapid tumor penetration, good solubility and improves stability. In preclinical studies, CD19xCD3 (Blincyto^TM^, Amgen, Breda, Holand) and CD19xCD16 bsDbs exerted a synergistic effect in non-Hodgkin’s lymphoma [47]. Various modifications to these recombinant bispecific molecules have been developed to improve their stability and affinity. For example, an additional middle linker has been used to join two fragments of polypeptide chains, joining all domains in a single polypeptide, thereby forming Single-chain Diabodies (ScDbs) (Figure 1) [48]. In preclinical studies, scBsDb CD3xPSCA proved to be effective in prostate cancer cells [49]. Finally, the tandem scFvs (TaFvs) was developed (Figure 1). In this case, two scFv molecules were linked by a short linker to give a very flexible structure. Anti-PSMAxCD3 scBsTaFvs antibodies stimulate T cells to eliminate prostate cancer cells [20].

A major achievement was the use of tandem bispecific T-cell engager molecules (BiTE^®^, Amgen, Southend Oaks, CA, USA) consisting of an anti-CD3 domain and an anti-tumor-associated antigen (TAA) connected by a short peptide linker (Figure 1). The advantage of the molecules produced by the BiTE technology is a significant reduction in the intercellular space between the lymphocyte linked to this antibody and the neoplastic cell, which facilitates the formation of an immune synapse and the activation of lymphocytes without MHC class I participation [50]. After binding to the neoplastic cell, the BiTE antibody attracts T lymphocytes to this target cell, stimulating them to form adhesins and cytolytic substances such as granzyme, perforin and cytokines [51]. Neoplastic cell death may also occur through the activation of caspases or death receptors [52]. Within the synapse, a junction is made between two cells via the adhesins ICAM-1 on the tumor cell and LFA-1 on T cells [53]. The TCR and MHC-I receptor do not participate in the interaction between these cells. The first BiTE antibody introduced into clinical practice was blinatumomab [54]. In 2014, the FDA granted blinatumomab breakthrough therapy status and is implementing an accelerated registration procedure for the treatment of relapsed or refractory Philadelphia chromosome-negative cellular acute lymphoblastic leukemia (ALL) with CD19 expression, indicating that the drug may be more effective than other standard drugs used in indicated cases [55]. The use of this antibody also caused fewer side effects than conventional treatment [56]. The complete remission (or cRh) rate with blinatumomab in Phase 2 studies was 69%. This molecule binds to the CD19 receptor on leukemic cells and to the CD3 receptor on T lymphocytes [23]. Due to the fact that the CD19 antigen is present on most B-type ALL leukemia cells and is absent on normal plasma cells and on hematopoietic stem HSCs, it is a very good therapeutic target [56]. The examination of the cells of patients treated with blinatumomab showed that their number of T lymphocytes increases, which lead to the lysis of B lymphocytes, including cancer-transformed CD19 [56]. Within a few days after starting blinatumomab therapy, the number of B lymphocytes falls below detection [57]. This condition persists throughout the administration of this drug. The advantage of blinatumomab is its anti-cancer effect at low doses [58]. As an adverse effect, it may rarely induce a cytokine storm or neurotoxicity, such as other BsAb [59,60]. Blinatumomab is also used in people with minimal residual disease after B-cell ALL treatment.

There are currently many BiTE-type molecules in preclinical and clinical research. An example is pasotuxizumab AMG 212/BAY 2010112 recognizing CD3 and prostate-specific membrane antigen (PSMA), highly expressed in prostate cancer cells and not expressed on normal cells [61]. Clinical studies of this antibody indicate that it has an acceptable safety profile and shows dose-dependent anti-tumor activity and has managed to achieve long-term response in two cases. Thus, evidence has been obtained that BiTE can be effective in solid tumors [62].

Bispecific and Trispecific Killer Cell Engagers, BiKE and TriKE antibodies, respectively, that kill cancer cells without prior sensitization also seem to be future-oriented. An example is the CD16/CD19/CD22 antibody, which binds two target molecules, CD19 and CD22 on a tumor-transformed B-cell, which enhances the specificity of its action [63,64].

Various modifications of the BiTE molecules have contributed to the improvement of their pharmacokinetics. Due to their small size and the lack of an Fc fragment, they show better and faster tumor penetration than antibodies with the classical structure of the IgG molecule [65]. A problem with the use of small antibody molecules may be the short serum half-life of 2 to 4 h, leading to reduced uptake by tumor tissue and requiring continuous intravenous infusion [66]. Therefore, modified BiTEs with extended half-life (HLE) have been developed, which are canonical BiTEs fused to an Fc domain with an activity similar to canonical BiTE [67]. Thanks to this modification, the half-life of a single dose of HLE BiTE is 210 h, which allows the drug to be used once a week [67].

Another modification of the second-generation bispecific molecules is the dual affinity retargeting (DART) technology (Figure 1). It is a type of diabody with two non-covalently linked polypeptide chains. A modification in this case is the introduction of a C-terminal disulfide bridge between two VH subunits, which ensures structural stability. In vitro studies showed that the anti-CD19 and CD3 DART antibody was more effective than BiTE targeting the same antigens. The DART^®^ format was found to cross-link T and B cells more efficiently than BiTE [68].

## 4. Targeted Immunotherapy Based on Genetically Modified T Cells

Among the different types of adoptive cell transfer (ACT), CAR T cell therapy is the most advanced. Thanks to modern methods of genetic engineering, we can obtain custom defense cells programmed to fight specific cancer cells. The recombinant T-cell receptor (TCR) that is introduced into T cells via viral vectors is specific for the patient’s cancer antigens. The reprogrammed T cells with the chimeric antigenic receptor (CAR T) obtained in this way search for and attack tumor cells.

**Figure 2:**
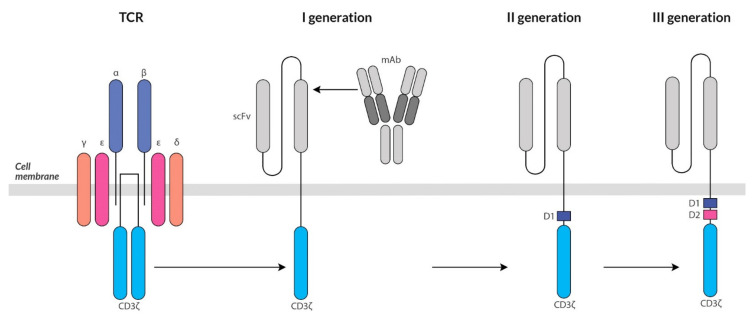
Structure of the first, second and third generation chimeric antigen receptors (CAR) constructed from the variable scFv fragment of a monoclonal antibody and from the cytoplasmic CD3ζ fragment of the T lymphocyte receptor (TCR). D 1 and D2—costimulatory domains.

CARs contain a single-chain Fv domain fragment for the tumor antigen, a membrane fragment of the CD3ζ receptor and a costimulatory moiety of the T cell receptor [69]. These interconnected molecules are expressed in the CAR T cell membrane. The costimulatory molecules are, e.g., CD28, 4-1BB, OX40, ICOS, NKG2D, DAP10 and 2B4 (CD244) (Figure 2) [70]. Recombinant receptors recognize the tumor antigen independently of MHC class I.

First generation CAR T cells lacking costimulatory molecules did not expand sufficiently after CAR stimulation [70]. Only the creation of the second-generation CAR T cells with attached costimulatory molecules were capable of both the cytotoxic effect and expansion [71]. Additionally, in the third generation CAR, two domains of costimulatory molecules are turned on, which should result in the enhancement of the stimulating effect of CAR T lymphocytes [72]. The solutions used in the third generation in practice give ambiguous results in terms of the effectiveness of their operation compared to the second generation [73]. The reasons for the observed effect are as yet unclear. High therapeutic efficacy was obtained when using two signaling domains (CD28 and CD3z) in combination with the 4-1BB ligand [73]. Such a combination of signaling domains allows for a balance between duration and antitumor activity, which was accompanied by an increase in the CD8/CD4 ratio [74]. Thanks to the latter modification, CAR T cells are not only programmed to destroy specific neoplastic cells but also have a longer life span [75].

Side effects that may arise from this therapy include tumor lysis syndrome or cytokine storm, the effects of which, however, can be eliminated using the anti-interleukin-6 receptor antibody tocilizumab [76]. Neurotoxicity, a decrease in the number of normal B lymphocytes in the case of CAR-T (CD19) therapy, hypotension and tachycardia may also occur. Sometimes, the side effects are so severe that they require intensive care, and rare cases of death have been reported [77]. An elimination gene such as the truncated form of epidermal growth factor receptor (EGFRt) has been developed to prevent serious side effects. EGFRt cannot bind to its natural ligand but has the ability to bind cetuximab, which triggers CAR T cell death by a variety of mechanisms [78]. Currently, other mechanisms are being worked on for the regulation of CAR T cells’ function, trying to use the switch ON/OFF strategy [79]. This developed system allows you to control the activity of CAR T cells by deliberately turning them off in the event of a threat and turning them back on when the threat is resolved. A method developed and tested in mice is to administer a compound that turns off the CAR-T cell when it binds to an antigen on a tumor cell. This effect disappears when the administration of the compound is discontinued, allowing cells to resume anti-tumor activity [80,81]. Other modifications of CAR T cells consist, for example, in switching on suicidal genes such as inducible caspase 9 in order to eliminate these cells after fulfilling their role in the body [81,82].

In 2017, the FDA approved the first cell-based gene therapy for the treatment of refractory, relapsed B-cell precursor ALL in children and young adults. The genetically modified cells are the patient’s own T cells into which genes for the chimeric antigen receptor, in this case, directed to the CD19 surface antigen on leukemic cells, are inserted [75]. Complete remission was achieved in approximately 90% of patients with relapsed or refractory ALL, as well as failure after bone marrow transplantation. Permanent remission has been followed for up to 24 months [83]. The advantage of this therapy is that CAR T cells proliferate in the patient’s organism up to 1000 times more than that implanted in the patient [75]. They have been detected in the blood, bone marrow and cerebrospinal fluid of patients [84]. In addition, persistent CAR T cells may characterize immune memory. This treatment approach shows promise in relapsed leukemias. The efficacy of CAR T cells therapy targeting the CD19 antigen has also been demonstrated in highly resistant and massive chronic lymphocytic leukemia (CLL) disease [85]. A meta-analysis conducted on the results of studies published between 1991 and 2014 showed that CAR T CD19 therapy gave the highest response rate in patients with ALL (93%, 95% CI: 65–100%) compared to patients with CML (62%, 95%). % CI: 27–93% and indolent B-cell lymphomas (36%, 95% CI: 1–83%) [85]. The analysis also confirmed the high response rate to treatment with CD19-CAR T cells in treatment-refractory B-cell tumors. Importantly, the analysis showed that the key factors for a better clinical response were lymph reduction and not administering IL-2 to patients [86].

So far, CAR T cell therapy has been successfully used only in hematological neoplasms [87]. So far, an unsolved problem is the effective treatment of solid tumors with this method. The reason for this is the lack of identified tumor-specific antigens that would be highly expressed and present on majority neoplastic cells. Antigens that are overexpressed on some neoplastic cells are also present at low concentrations on normal cells, which can lead to high toxicity [88,89]. Solid tumor cells are much less homogeneous in the expression of a known specific antigen compared to hematological CD19 + tumors. There is a need to search for tumor-associated antigens that could be a suitable therapeutic target. The hope is that neoantigens, which are highly immunogenic and are not present in normal cells, arise in neoplastic cells as a result of mutations or, less frequently, are derived from oncogenic viruses [90], whose presence is still being investigated. The search for neoantigens with appropriate traits useful in treatment, such as an appropriate amount and expression in the changing environment of the tumor, may become the target of personalized therapy in the future [91].

A serious problem is the limited access of CAR T cells to neoplastic cells due to the specific tumor microenvironment. One of the many ways to overcome this obstacle may be to stimulate CAR T cells to produce and secrete heparanase (HPSE). CAR T cells appear to be deficient in the ability to produce HPSE and therefore cannot efficiently degrade the extracellular matrix in a tumor [91]. Modifying CAR T cells to produce HPSE enables them to degrade ECM and improves tumor mass infiltration and enhances antitumor activity [92]. Moreover, tumor-associated stromal cells secrete indoleamine 2,3 dioxygenase (IDO) which contributes to acidification of the microenvironment and reduces the anti-tumor activity of CAR T cells [93,94] and promotes the induction of Treg lymphocytes. A serious problem is the immunosuppressive environment of the tumor, which consists of adenosine-PGE2 signaling, contributing to a strong inhibition of proliferation and activation of T effector lymphocytes, NK and TAM cells and promotes the activation of Treg, creating favorable conditions for tumor growth [95]. Other immunosuppressive agents in the tumor microenvironment that may be therapeutic targets are IL-4 and IL-10 [96,97]. Additionally important for the therapy would be the inhibition of TGFbeta secreted by both tumor cells and tumor associated cells.

Ways to increase the efficacy of CAR T cells in solid tumors, inter alia, by enhancing the migration of these modified cells to the tumor, improving survival and proliferation, improving specificity, bypassing the immunosuppressive environment and reducing the side effects of cytokine storms are discussed in an extensive review [98]. On the other hand, TCRs rather than CARs seem to be more useful in the recognition of neoantigens due to the fact that the former can recognize many more antigens, also processed intracellularly [99]. By contrast, CAR T cells can only recognize extracellular antigens. On the other hand, a limitation in TCR therapy is the downregulation of MHC on tumor cells necessary for antigen recognition by TCR.

The use of autologous tumor infiltrating lymphocytes (TILs) is also considered as a type of immunotherapy [99]. Phase II clinical trials have shown that half of patients with metastatic melanoma showed an objective response (OR) after TILs application [100]. This therapy has proved ineffective in non-immunogenic tumors due to the low amount of polyclonal tumor-specific T cells obtained after ex vivo expansion [97]. Increasing its effectiveness in treatment is associated with the improvement of the technique of isolation of these cells from tumor tissue [101] using appropriate markers, e.g., co-stimulatory surface receptor CD137 (4-1BB), which appears on T lymphocytes with a high capacity to recognize and respond to neoplastic cells [102].

Research also aims to develop bispecific CAR T cells for CD19 and CD22 simultaneously for the treatment of refractory and relapsed ALL [102]. Although most cells in B-ALL have the CD19 antigen, there are cells lacking this antigen and responsible for frequent relapses. CD19/CD22 CAR T cells would prevent these relapses and lead to a further improvement in the treatment efficacy of this leukemia. Based on the first phase of clinical trials, it is already known that improvement was also obtained, but the problem of the presence of blast cells with negative or low expression of these two antigens has not yet been solved [103]. A problem with this downregulation of CD19 and CD22 is their endocytosis after crosslinking [104,105]. A way around the obstacle is to use the Lym-1 epitope of the HLA-DR antigen, which is not endocytosed after cross-linking [106] and is highly expressed on most human leukemia B cells [105]. Additionally, to improve the expansion, a new DAP signaling domain was used instead of the classic 4-1BB and CD3z (BB3z) [107]. Thus, huLym-1-B DAP CAR T cells were obtained, which, in ex vivo and in vivo tests, has better expansion and shows higher cytotoxicity towards tumor cells. The new huLym-1-B DAP CAR T cells therefore seem to be a promising therapy worth further research.

The CAR-NK therapy cannot be ignored, as it may be more effective and safer than CAR T cells. NK cells use the Natural killer group 2D (NKG2D) receptor to recognize neoplastic cells. It appears that, in vitro and in vivo, the NKG2D transduction of activated and expanded (NKAE) NK cells results in an increase in their antitumor activity in comparison to autologous NKG2D-CAR CD45RA T cells [108]. CAR-NKAE cells also performed very well in toxicity tests, as they showed no toxicity to autologous PBMCs [107].

## 5. Oncolytic Viruses

The idea to use viruses to fight cancer first appeared several decades ago. However, only the development of genetic engineering made it possible to implement the original plans. Oncolytic viruses are a type of immunotherapy designed to selectively attack and kill cancer cells and enhance anti-cancer immunity [108]. Neoplastic cells infected with an oncolytic virus return under immunological surveillance, as they begin to express major histocompatibility complex class I (MHC I) molecules on their surface [109].

Research on oncolytic viruses uses both naturally occurring oncolytic viruses and modified viruses in such a way that they have an affinity only for neoplastic cells [110,111]. Viruses with natural tropism to neoplastic cells include natural oncolytic viruses are reoviruses, Newcastle disease virus (NDV) and vesicular stomatitis virus (VSV) [112]. Modified oncolytic viruses are altered so that they can be picked up selectively only by mutant tumor cell receptors, or a deletion is used that allows the virus to selectively replicate in tumor cells [112,113,114].

The advantage of oncolytic viruses is also their ability to replicate in neoplastic cells, thanks to which they can infect other cells [112,115]. Moreover, if they infect normal cells, they have reduced pathogenicity in these cells [116]. The most commonly modified viruses for anti-cancer therapy are adenoviruses, vaccinia (vaccinia) and HSV [115,116,117]. In the treatment of patients with advanced, unresectable melanoma without metastases to internal organs the oncolytic virus talimogene laherparepvec—T-VEC—is powerful [118]. T-VEC results from the modification of HSV-1, for example, two viral genes were deleted: the ICP34.5 gene, in order to prevent neurovirulence and improves viral selectivity in against neoplastic cells, and the ICP47 gene, which blocks the presentation of viral antigens by infected cells to CD8 T cells [119]. By deleting the ICP47 gene, the immune response against infected tumor cells is enhanced. The FDA approved the T-VEC based on studies that confirmed the overall durable response rate of 16.3% of patients, compared with 2.1% of the reference group treated with GM-CSF [120]. The drug is well tolerated and may cause mild to moderate flu-like symptoms.

Strategies that are used in the creation of oncolytic viruses rely on the deletion of specific genes, such as genes essential for the replication of the virus in normal cells. An example is the deletion of genes responsible for inactivating cell suppressor genes, such as the p53 or rb gene, so that the cell cycle does not stop after infection and the virus replicates freely in the cell [121,122,123]. Viruses with such a defect can replicate only in tumor cells with mutations that inactivate these genes. Another strategy is to replace the viral promoter with a tissue-specific one to limit viral replication to cells expressing specific antigens, e.g., hypoxia-induced factor 2 (HIF-2) or prostate-specific antigen (PSA), which are overexpressed in certain types of cancer cells [124,125].

With the use of viruses, “suicidal genes” are introduced which sensitize neoplastic cells to a cytostatic, e.g., thymidine kinase gene, purine nucleoside phosphorylase, cytochrome p450, cytosine deaminase [126]. The introduced enzymes convert the inactive substance into a cytotoxic drug whose activity is limited to the infected tumor cells. In the case of the T-VEC virus, a gene for GM-CSF was introduced to improve the anti-tumor response by activating dendritic cells. This strategy increases the presentation of tumor antigens, which leads to the activation of cytotoxic T lymphocytes and stimulates the immune system to a systemic response, leading to the elimination of distant metastases [127].

**Figure 3:**
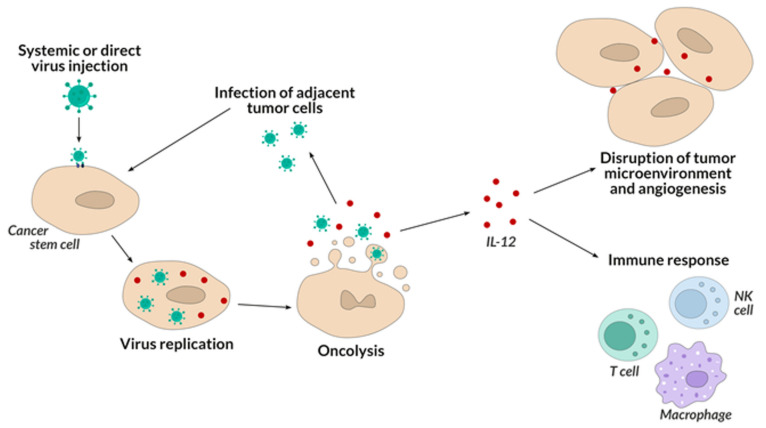
The effects of oncovirus therapy. Oncolytic viruses selectively replicate and lyse cancer cells compared to normal cells which lack these effects.

Another advantage of HSV-1 oncolytic viruses is the stimulation of cancer cells to secrete IL-12, which cytokine supports the immune response against persistent cancer cells, which increases the anti-cancer effect of the therapy (Figure 3). Elevated levels of IL-12 can also exert an anti-angiogenic effect by inhibiting the production of new blood vessels that allow cancer cells to grow and differentiate.

However, the problem in this therapy is the reaction of the immune system to the antigens of the oncolytic viruses themselves, which leads to their neutralization [128]. This is prevented by the use of carrier cells, e.g., T lymphocytes, which can deliver the oncolytic virus to the neoplastic cells, preventing them from being neutralized earlier [129].

Clinical trials are currently underway on a number of oncoviruses with potential application in cancer therapy such as, e.g., GL-ONC1 Vaccinia oncolytic virus; LOAd703 oncolytic adenovirus administered to patients with pancreatic cancer; or ADV/HSV-tk oncolytic therapy involving the thymidine kinase expression of the herpes simplex virus with adenovirus and valaciclovir therapy in patients with triple-negative metastatic breast cancer and small cell lung cancer (NSCLC) metastases. In Japan, several oncolytic viruses have been developed from 2018 to date, some of which have been introduced into clinical trials. An example is G47Δ, a third-generation oncolytic virus of HSV-1, included in Phase I and II clinical trials against neoplasms such as glioblastoma, prostate cancer and olfactory neuroblastoma [130]. Three forms of it have entered clinical trials: HSV1716 showing clinical efficacy in high-grade glioma, as well as in solid brain tumors of pediatric patients [131], G207 in recurrent malignant glioma [132] and self-replicating herpes virus—MO32 recommended especially in progressive, malignant glioma [133] or G47Δ (with an additional α47 deletion attached to G207) as the only third-generation HSV-1 showing clinical efficacy in Phase I and II clinical trials in patients with recurrent glioblastoma, prostate cancer or mesothelioma approved for use in 2016 in Japan by the Japanese Pharmaceuticals and Medical Devices Agency (PMDA).

**Figure 4:**
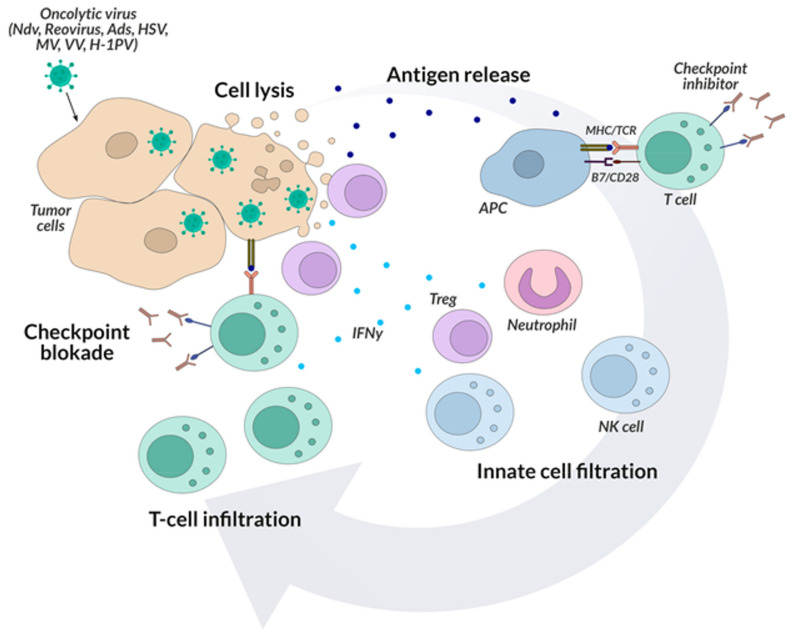
The diagram shows combining oncolytic virus therapy and immune checkpoint inhibitors. As a result of the action of oncolytic viruses, neoplastic cells are lysed and the immune response is induced, and thanks to the use of checkpoint inhibitors, the immune defense of the organism is strengthened. Ndv—Newcastle disease virus, Ads—Adenovirus, HSV—Herpes simplex virus, MV—Measles virus, VV—Vaccinia virus, H-1PV—H-1 protoparvovirus.

Despite the admiration of the scientific world, the response from the human body affected by cancer to the introduction of an oncolytic virus into its organism is still usually ineffective. On the one hand, it is hindered by the tumor’s protective mechanisms and its diverse phenotype, and on the other hand, the introduction of an oncolytic virus into the body stimulates the host’s immune system to respond, thereby limiting the replication of the virus [134]. Hence, the combination immunotherapies aimed at multiple goals seem promising. For example, introducing an oncolytic virus prior to tumor removal surgery may alter the body’s immune response and enhance the effects of subsequent checkpoint inhibitor treatment. The current research interests are focused on combined cancer treatment [135]. Hence, the current clinical trials using oncolytic viruses in cancer therapy emphasize the importance of combining them with checkpoint inhibitors, which leads to the enhancement of the final effect by simultaneous elimination of cancer cells and dramatic changes in its microenvironment. It seems important here—as in the case of bispecific antibodies—to develop a technology of combining various types of viruses with checkpoint inhibitors (Figure 4).

Preclinical and clinical data show that oncolytic viruses can induce anti-tumor immunity and significantly increase the infiltration of immune cells (including CD8 + cytotoxic lymphocytes) into the local tumor microenvironment. The influx of viral infection changes the tumor microenvironment, resulting in the continuous activation of many different cells of the immune system and a cascade of proinflammatory cytokines which is a target for checkpoint inhibitors as they are most effective in environments with large lymphocytic infiltration [136,137]. An example of a clinical trial that combines oncolytic viruses with checkpoint inhibitors is CAPTIVE CAPRA, in which patients with advanced melanoma were injected with multiple CVA21, with multiple doses of pembrolizumab [138]. The effectiveness of the proposed combination therapy was 73%, which seems to revolutionize combination therapy in many types of cancer. In other studies, the combination of T-VEC with ipilimumab in patients with inoperable stage IIIB-IV melanoma in Phase Ib/II studies (NCT01740297) was more effective than ipilimumab alone and was well tolerated [139]. Another example of high effectiveness seems to be the aforementioned HF10 polytherapy with ipilimumab, an anti-CTLA-4 checkpoint inhibitor in patients with inoperable or metastatic melanoma (in 2014 in the USA—NCT02272855; in 2018 in Japan—NCT03153085).

## 6. Cancer Vaccines

Tumor vaccines aim to stimulate an immune response against tumor associated antigens (TAAs) or tumor strand associated antigens (TSAAs) after immunization with purified, recombinant or synthetically produced epitopes.

Various methods are used to expose the patient’s immune system to tumor antigens. Hence, attempts are being made to develop peptide (protein/peptide) anti-tumor vaccines, DNA- or RNA-based genetic vaccines and whole tumor vaccines based on dendritic cells [140,141,142,143,144,145] (Table 2). In the case of genetic vaccines, DNA or RNA for TAAs is introduced via a plasmid or viral vector [146,147].

A problem with DNA-based vaccines may be the risk of insertional mutagenesis [146]. In this regard, RNA vaccines may be safer. Additionally, they are present only transiently in cells due to enzymatic degradation. RNA is obtained from autologous tumor cells, and tumor cRNA libraries are produced, which provide a large amount of tumor RNA [148]. RNA therapy allows the exposure of tumor cells to a variety of tumor antigens TAAs and TSAAs [149]. The latter advantage is important in the context of the relationship between the tumor stroma and tumor cells in the anti-neoplastic response [143,150]. However, the efficacy of the anti-cancer genetic vaccine has not been demonstrated in clinical trials [151,152]. The problem is the instability of RNA and therefore low efficiency, that is why, so far, DC cells have been exposed to neoplastic RNA ex vivo, by electroporation [153], the use of nanoparticles [154], liposomes or synthetic polymers [155]. One of the developed DNA vaccines, which was administered to DC by electroporation, showed increased regression of cervical neoplasia in the phase 2b clinical trials [156].

Currently, various methods of mRNA stabilization have been developed, including the creation of analogous mRNA caps, which extend the duration of this molecule in the body, increase the level of protein expression in dendritic cells several dozen times and allow it to be used directly in vivo [157,158,159]. The modification consists in replacing one oxygen atom with a sulfur atom (beta S-ARCA analog) or with a BH3 group (beta B-ARCA analog) in the triphosphate bridge cap 5. In this way, the duration of the mRNA molecule in the organism is tripled. The affinity of mRNA to the factor initiating the biosynthesis of the eIF4E protein also increases. Therefore, it can be expected that there will be a new generation of mRNA anti-cancer vaccines that will have positive results in clinical trials.

In the case of peptide vaccines, the quality of the peptide used is of importance. Not only is the selection of the appropriate peptide important but so is the length of this molecule, which must be optimal to induce an immune response [156]. In order to increase the immune response to vaccines, attempts are made to use immunoadjuvants such as GM-CSF or interleukin-2 or toll-like TLR agonists [160]. Cytokines enhance the immune response to cancer vaccines by stimulating dendritic cells, NK and T cells. The basis of the action of cancer vaccines is the presence of activated DC cells capable of presenting the antigen. It is known that, in neoplastic disease, the phenomenon of immunosuppression leads to the inhibition of DCs function [161]. Therefore, cancer vaccines, especially peptide vaccines, are often combined with immunoadjuvants, which are primarily used to unblock DCs. Following such stimulation, DCs express on their surface not only vaccine antigens but also costimulatory molecules that are essential for T cell activation and migrate to lymph nodes [162]. TLR agonists are adjuvants that strongly stimulate DC cells in clinical trials [145,163].

To date, peptide vaccines have shown limited efficacy in clinical trials. The reason for these failures is the intracellular processing of peptide molecules, which negatively affects antigenicity. The tumor microenvironment, which promotes immunosuppression, should also be taken into account. A solution to this problem may be the simultaneous use of several therapies with different goals, e.g., combining peptide vaccines with adjuvants, cytostatics and immune checkpoint inhibitors.

So far, only one dendritic-cell-based cancer vaccine (PROVENGE^®^, Dendreon Corporation, Seal Beach, CA, USA) has been approved by the FDA for therapy and is used only in the USA for the treatment of castration-resistant prostate cancer [164]. Apart from the huge cost of this therapy, its effectiveness is not high, as it improves the median survival by an average of 4.1 months compared to placebo (25.8 vs. 21.7 months) and does not affect the time of progression [165]. The dendritic cells in this vaccine are derived from the patient’s PBMCs and are incubated with the PA2024 fusion protein consisting of prostate acid phosphatase and GM-CSF. Its advantage is good tolerance. Little effective dendritic vaccines are being combined with checkpoint inhibitors or oncolytic viruses. Inhibition of immune checkpoints may increase the immune response to the vaccine. If the synergism of anti-cancer activity was achieved, it could be a promising direction of therapy. An example is the combination therapy of MG1-MAGEA3 oncolytic virus with Ad-MAGEA3 vaccine and pembrolizumab targeting non-small cell lung cancer of patients who have undergone 1 course of standard cisplatin chemotherapy and at least one treatment with an antibody targeting programmed cell death 1 receptor (anti-PD-1, e.g., pembrolizumab) or (anti-PD-L1) (in 2017 in Canada—NCT02879760) [166]. Another good example is the recombinant carcinoma-induced ovarian cancer (CEA) vaccine containing measles virus (MV-CEA) or measles oncolytic virus encoding sodium thyrotropic symporter (MV-NIS), recommended for the treatment of patients with progressive, relapsed or refractory treatment of ovarian or primary peritoneal cancer (under recruitment at Mayo Clinic—NCT00408590). Another example of a vaccine that is part of a combination therapy that has seen the light of day with the possibility of introduction in humans is MG1-MAGEA3 with Ad-MAGEA3 and pembrolizumab (composed of: Ad-MAGEA3—an adenoviral vaccine expressing melanoma-associated antigen 3 (MAGEA3), a tumor antigen; MG1-MAGEA3 MG1 Maraba oncolytic virus expressing melanoma-associated antigen 3 (MAGEA3), a tumor antigen; Pembrolizumab—monoclonal antibody; PD1 checkpoint inhibitor; chemotherapy with cyclophosphamide) recommended for patients with previously treated metastatic melanoma or cutaneous squamous cell carcinoma (Pelican) not yet recruited but planned at Turnstone Biologics, Inc.—NCT03773744. MG1-E6E7 vaccine with Ad-E6E7 and Atezolizumab recommended in patenty with HPV-related cancers (recruitment from December 2018 in Florida, University of Miami—NCT03618953). The problem with the effectiveness of genetic vaccines may be also the lack of identified appropriate tumor antigens that could induce a strong and specific immune response.

## 7. Research on Improving the Effectiveness of Biological Therapies

### 7.1. Searching for Neo-Antigens

Tumor-associated antigens (TAAs) used to develop immunotherapy did not bring the expected results in solid tumors. The reason for these failures may be the poor recognition of these antigens on tumor cells. Moreover, their presence also on normal cells can generate severe side effects in healthy tissues, as has been observed in CAR T cell therapy [167,168].

Tumor antigens to be targeted by new biological therapies for solid tumors should meet certain conditions. They should be unique to neoplastic transformed cells and be expressed on most neoplastic cells, should not be subject to the mechanism of central immune tolerance and should induce a specific and strong immune response when recognized as foreign antigens [169].

Therefore, recent research on the search for antigens for biological therapies focuses on neoantigens/neoepitopes (tumor specific antigens, TSAs), which arise in neoplastic cells as a result of mutations or epigenetic phenomena, leading to the formation of an altered protein with a unique structure, characteristic for a given patient [170]. The advantage of neoantigens is that they are not presented in the thymus and therefore are not immunocompromised. The strategy of using neoantigens is the path to personalized therapy. Much progress in the search for such antigens is possible thanks to the development of the NGS technique, which allows the detection of all mutations in a tumor. Clinical trials provide arguments for the use of neoantigens. The use of neoepitope-specific TILs as part of adoptive T target therapy has shown positive therapeutic results in clinical trials [171]. It has been shown that the administration of neoantigens in the form of a vaccine results in the stimulation of neoantigen-specific T lymphocytes [172,173].

### 7.2. Tumor Stroma-Associated Antigens as a Target of Anticancer Therapy

Tumor stroma-associated antigens (TSAAs) are also a subject of research for future cancer therapies, as they occur on normal cells that can support tumor cells in their proliferation, support the process of metastasis, participate in tumor angiogenesis [174]. Tumor stroma-associated cells overexpress specific TSAAs [175]. One can also observe functional characteristics different of these some cells in normal tissue [176]. Tumor-associated fibroblasts overexpress fibroblast activation protein α (FAPα; seprase). FAPα overexpression has been shown to correlate with increased tumor development [177]. The proteins produced by endothelial cells within a neoplastic tumor are TEM1 and TEM8 [177,178]. Other proteins that are upregulated in the tumor microenvironment are the matrix metalloproteinases (MMPs) secreted by stromal cells such as fibroblasts and endothelial cells, which assist tumor cells in producing these proteins essential for extracellular matrix remodeling [179]. TSAAs also includes PSMA, secreted not only by prostate cancer cells but also by the stoma endothelial cells of some types of carcinomas, such as kidney, bladder, breast and non-small lung cell carcinoma [180].

### 7.3. Universal Therapeutic Goals

**Figure 5:**
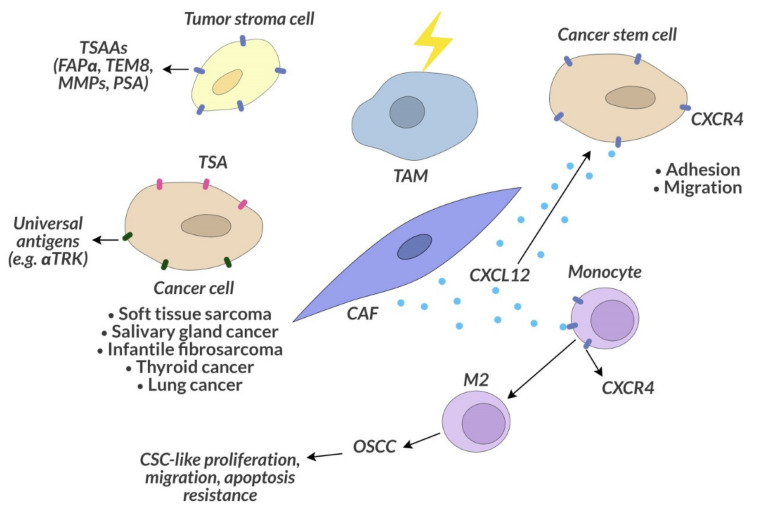
Diagram showing potential therapeutic targets on cancer cells. TSA—Tumor-specific antigen, TSAAs—Tumor Stroma-Associated Antigens, FAPα—Fibroblast activation protein α, MMPs—Matrix metalloproteinases, TEM 8—Tumor endothelial marker 8, PSMA—Prostate-specific membrane antigen, TRK—tyrosine kinase, TAM—Tumor-associated macrophage, CAF—Cancer-associated fibroblast, M2—Macrophage 2, CXCL12—C-X-C motif chemokine 12, CXCR4—chemokine receptor for CXCL12, OSCC—Oral squamous cell carcinoma, CSC-like—Cancer stem-like cell.

Currently, the trend in cancer therapy research is the search for a universal biological therapeutic target for various types of cancer. An example of this strategy is the FDA-approved accelerated drug Vitrakvi (larotrectinib) in 2018, which targets the key cancer genetic driver, present in various types of cancer, the neurotrophic receptor tyrosine kinase (NTRK) gene fusion. The NTRK genes encode the transmembrane receptor proteins TRK A, B and C [181]. It is expressed in nervous tissue, and the physiological role of this receptor is to regulate the development and function of the human nervous system [182]. In cancer cells, NTRK genes can be fused with other genes, and the resulting fusion genes act as constitutively activated kinases, overexpressed and promoting tumor growth [181]. This mutation is rare but is present in various types of cancer, such as mammary analogue secretory carcinoma, cellular or mixed congenital mesoblastic nephroma, soft tissue sarcoma, salivary gland cancer, thyroid cancer, lung cancer and infantile fibrosarcoma (Figure 5). Vitrakvi gave a durable overall response rate in 75% of people with various types of treatment-resistant solid tumors. In 73%, this response lasted for at least 6 months [183].

Other therapeutic targets common to various cancers are also being sought. Such therapies are becoming possible thanks to the growing knowledge of the basics of cancer cell biology. The LPCAT1 enzyme, under the influence of which there are changes in the lipid composition of the cancer cell membrane, may be an effective target in the future. The activity of this enzyme is enhanced in many types of cancer cells, it stimulates tumor growth and is important for its survival [184].

### 7.4. Cancer Stem Cells

One of the major problems in the fight against cancer is that cancer stem cells can survive treatment by slowly dividing, being resistant to cytostatic drugs and escaping the immune system. Until we deal with cancer stem cells, we will not be able to effectively treat this disease.

Finding the therapeutic targets of key importance for managing the behavior of cancer stem cells among a multitude of different pathways could effectively block the development of the disease. The example of CML stem cells shows that in these cells, unlike in CML progenitor cells, there is cooperation between BCR-ABL and many growth factors, tumor suppressors, as well as factors that govern the quiescence and maintenance of CML stem cells [185]. The co-operations of various factors with BCR-ABL modulate the signaling of this fusion protein and lead to resistance to TK inhibitors. Epigenetic modifiers and metabolic reprogramming of stem cells and the role of microRNAs in their survival should also be taken into account [186]. Another approach is to identify tumor-specific surface markers for individual cancer stem cells and to develop monoclonal antibodies for them [187]. The listed exemplary concepts of new therapeutic strategies represent a rather distant perspective in cancer therapy.

Nanostructured toxins can also be an effective alternative. An example of such a therapeutic strategy under development is the combination of a ligand for the CXCR4 receptor with diphtheria toxin (DITOX) and the Pseudomonas aeruginosa exotoxin (PE24) molecules, which are ribosome-inactivating proteins (RIPs) that inhibit the eukaryotic elongation factor 2 (eEF-2). They were applied to colorectal cancer stem cells overexpressing the CXC4 chemokine receptor, which is involved in drug resistance. These cancer cells administered to mice were successfully killed after the administration of this therapy [188]. This toxin caused the death of apoptosis-resistant cells by pyroptosis.

One important factor that manages the stemness of CSCs in renal cancer is the Notch pathway [189]. Blocking Notch 1 and 2 with inhibitors leads to the loss of stem cell characteristics, including self-renewal, migration, invasiveness and chemoresistance and the ability to induce cancer in vivo. In contrast, overexpression of Notch1 leads to an increase in CXCR4 expression and an increase in the C-X-C-induced chemokine 12 (CXCL12) chemotaxis of renal cancer CSCs.

### 7.5. Cancer Microenvironment

An important therapeutic strategy is to influence the tumor microenvironment, which is essential at every stage of tumor development. It protects stem cells from chemotherapy and participates in the regulation of quiescence. Normal cells with overexpression of specific proteins characteristic of the tumor microenvironment may also be a universal therapeutic target, as they support tumor growth.

CAFs and TAMs are particularly important components of the tumor microenvironment. These cells promote the proliferation of neoplastic transformed cells, invasion and metastasis [190,191]. By studying the microenvironment of the human oral squamous cell carcinoma (OSCC), CAFs have been shown to attract monocytes to the tumor by secreting the CXCL12 chemoattractant that binds to the CXCR4 monocyte receptor [192]. In response to this stimulation, monocytes differentiate into M2 macrophages and lead to the conversion of the OSCC-derived cell line into CSC-like cells, which increases their proliferation and migration and protects against apoptosis (Figure 5) [192]. The CXCL12 molecule produced by the bone marrow niche cells and its CXCR4 receptor on leukemic cells are also responsible for drug resistance in myeloid leukemia [193]. The use of the CXCR4 inhibitor sensitizes leukemic cells to chemotherapy and inhibits the homing of myeloid leukemia cells to the corresponding niches in the bone marrow [194].

Leukemic stem cells (LSCs) are associated with a microenvironment that contributes to their resistance to treatment and to initiate relapse. LSCs transform the microenvironment in the bone marrow to their advantage, making it unfavorable for HSCs, which causes these cancer cells to gain an advantage over HSC [195]. They are kept in a quiescent state and receive protection against chemotherapy from the microenvironment [196,197]. CXCR4 may be a new therapeutic target for many types of solid tumors and leukemias. Apart from the complex and not yet well-known interactions of the bone marrow microenvironment and LSCs, the role of growth differentiation factor 15 (GDF15) produced by CAFs also deserves attention [198]. Similarly, a decrease in the expression of the lumican gene in bone marrow mesenchymal stem cells (BM-MSCs) leads to the chemotherapy resistance of LSCs [199].

One should take into account that therapies targeting cancer stem cells can also affect normal stem cells due to the many similarities between transformed and normal cells. The design of therapies against cancer stem cells should target cancer stem cells as precisely as possible and should also overcome the conditions of a specific tumor microenvironment with poor vascularization and low oxygen levels not conducive to drug penetration [200].

### 7.6. Release of LSCs from a Natural Niche as a Therapeutic Strategy

In the case of leukemias or lymphomas, the method of elimination of cancer stem cells may be their mobilization into the peripheral blood, followed by isolation by modified leukapheresis combined with the capture of these cells with an antibody specific for their markers. The search for such markers is still ongoing.

A second strategy that is now being investigated is the mobilization of bone marrow stem cells, including LSC and HSC, into the bloodstream in combination with the use of chemotherapy. This strategy is based on the assumption that LSCs “torn” from their niche in the bone marrow can more easily enter the cell cycle and undergo chemotherapy-induced apoptosis.

Currently, strategies to disrupt the CXCL12/CXCR4 interactions that control homing and retention of LSC by administration of G-CSF are used to mobilize hematopoietic stem cells into the peripheral blood [201]. Improving the effectiveness of this strategy could contribute to the release of a large enough pool of LSCs to be removed from the body and obtain a therapeutic effect.

Many inhibitors are in development, such as plerixafor (AMD3100), an FDA-approved CXCR4 antagonist for the mobilization of HSC in combination with G-CSF. Plerixafor was tested in Phase I and II clinical trials (NCT01435343) in patients with relapsed and refractory AML in combination with G-CSF, fludarabine, cytarabine and idarubicin, achieving a high CR/CRi of 49%, of which 61% of patients underwent allogeneic HSC transplantation, but the OS after this treatment was only 9.9 months, and the DFS was 13 months [202]. More effective antagonists of CXCR4 are still being sought, and noteworthy is the synthetic peptide BL-8040, having a high affinity for CXCR4 [203]. In clinical trials in patients with AML (NCT01838395), BL-8040 peptide used as monotherapy strongly mobilized AML progenitor cells and induced their apoptosis. However, when combined with cytarabine, this peptide induced 38% CR and significantly improved OS in AML patients compared to cytarabine alone [204].

They also pay attention to other factors involved in LSC adhesion to the cells of the bone marrow microenvironment. An example is selectins, which are involved in the interaction between LSC and vascular niche [205]. The E-selectin antagonist GMI-1271 (uproleselan) mobilizes AML blasts into the circulating blood [205]. Promising results have been obtained in clinical trials (NCT02306291) in patients with relapsed and refractory AML treated with uproleselan in combination with chemotherapy (mitoxantrone, etoposide, cytarabine) [206]. The improvement in survival time and the occurrence of remissions in these patients correlated with high expression of E-selectin-ligand on AML blasts and on LSC.

Integrins, which are activated by CXCL12, also participate in the binding of CD34+ cells to ligands in the bone marrow niche [207]. The very late antygen-4 (VLA-4) is an integrin which plays a special role in AML, binding to fibronectin intercellular adhesion molecule 1 (ICAM-1) and vascular cell adhesion molecule 1 (VCAM-1). VLA-4 expression is associated with shorter survival in AML patients [208]. In addition, the use of anti-JAM-C antibodies inhibits HSC homing and induces their mobilization to the blood in mice [209].

Given the complexity of LSCs interactions with the microenvironment, acting on these cell populations by disrupting their interactions with the niche and inducing their mobilization into the bloodstream is still an open path to developing new therapies for AML.

### 7.7. The Problem of Cancer Cell Heterogeneity

The heterogeneity of neoplastic cells may contribute to the failure of treatment with inhibitors that target single molecular targets, despite the previous positive response of neoplastic cells to treatment [210]. An example would be NSCLC therapy with the third-generation EGFR inhibitor osimertinib. It has been observed that when osimertinib is administered to patients with EGFR mutant NSCLC who have previously received treatment with at least one line of therapy, a significantly shorter time to progression (PFS = 8.2 months) is observed [211] compared to the use of the drug in first lines of therapy (PFS = 18.9 months) [212]. It is presumed that this decrease in the effectiveness of osimertinib may be due to the increase in the heterogeneity of the population of cancer cells caused by the use of previous therapies [210]. This is an argument for not only the use of combination therapy but also for personalized treatment, modified at various stages of the disease.

## 8. Directed Enzyme Prodrug Therapy in Treatment of Cancer

An interesting strategy is the antibody-directed enzyme prodrug therapy (ADEPT) therapy being developed. The idea of ADEPT technology is related to the use of monoclonal antibodies that can be used as carriers of unique enzymes and binding specifically to tumors, where they can transform many prodrug molecules into potent cytotoxic agents within tumors [213]. This enables attaining higher drug concentrations than in the case of direct administration. Drug is thus produced extracellularly, and being a small molecule, it can diffuse through the mass of the tumor and also kill cells through transitive effect [214]. ADEPT is a less toxic chemotherapy for normal tissue and thus it can be combined with other methods, including immune therapy, in order to obtain better clinical benefits [215].

ADEPT using G2 (CPG2) carboxypeptidase, a bacterial enzyme isolated from Pseudomonas sp. [216], has been applied clinically, and it has no human analogue, catalyzing the degradation of reduced and non-reduced folates. Preclinical studies of CPG2 conjugated to non-internalizing antibodies targeting secreted tumor-associated antigens, such as human chorionic gonadotropin (hCG) and carcinoembryonic antigen (CEA), were performed in a mouse model with human choriocarcinoma xenografts-CMDA prodrug. They demonstrated complete or partial regression of tumor in the mouse [217,218].

The clinical trial uses CPG2 chemically conjugated to the F(ab)SD fragments of the murine anti-CEA A5B7 monoclonal antibody. In addition, another murine monoclonal antibody (SB43) targeting CPG2 has been developed. SB43 inactivates CPG2 and, to avoid the inactivation of CPG2 in tumors, it has been chemically galactosylated (SB43gal), thus it is quickly cleared from the circulation via carbohydrate receptors in the liver [219].

## 9. The Progress of Targeted Therapy

Current methods in research on anti-cancer drugs such as NGS and advanced computational methods allow for accelerating the rate in the search for small molecule targeted drugs, such as inhibitors of protein kinases or inhibitors of the mTOR pathway.

Protein kinases modify the activity of other proteins involved in various cell functions through a phosphorylation reaction. Impairment of their functions is observed in various neoplasms [4]. The therapeutic success achieved with the use of the mutant BCR-ABL kinase inhibitor mentioned in the introduction in patients with CML prompts research on inhibitors of other kinases involved in tumorigenesis. In search of protein kinase inhibitors, the first targeted therapy was developed in the treatment of AML with midostaurin in patients with the FMS-like tyrosine kinase 3 (FLT3) mutation, which occurs in 15–35% of patients with AML. There was a significant extension of OS compared to placebo [220]. Currently, inhibitors are tested for many protein kinases, including PIM serine-threonine kinases 1, 2 and 3. PIM kinases are overexpressed in AML and solid tumors such as colon cancer or prostate tumor, which is associated with poor prognosis [221,222,223]. Pan-Pim kinase inhibitors from the imidazopyridazine-thiazolidinediones group have been shown to exert anti-neoplastic activity in various neoplastic types in vitro and in vivo [224].

The advanced computational methods currently used make it much easier to precisely determine the structure of active sites for molecular targets, necessary to bind molecules and inhibit their activity or activation. In this case, computational techniques are helpful based on bioinformatics and cheminformatics, which deal with the development of databases and statistical algorithms that enable the analysis of data from biological and chemical research [225]. Thanks to them, it is possible to identify molecules that are the best drug candidates among many others. Molecular docking methods and ADMET research make it possible to analyze the interactions of these emerging candidate molecules with being the target of macromolecules and determine which of them have the best features of a potential drug [226].

## 10. Applications and Potential of Biological Therapies

Of the different biological therapies, recombinant antibodies have so far played the most important role in the treatment of cancer, some of which have proven to be breakthrough therapies such as checkpoint inhibitors. That is why such therapies are still intensively developed. The FDA has recently approved not only blinatumomab but also some other antibodies. The anti-EGFR/cMET antibody, amivantamab, approved by FDA in 2021 through an accelerated procedure, is intended for NSCLC patients with an EGFR exon 20 mutation in disease progression following platinum therapy [227]. The EGFR inhibitors available so far have not brought positive therapeutic results in patients with an exon 20 mutation, while, following amivantamab, the ORR was 40% and the mean duration of response (DOR) was 11.1 months. Treatment was discontinued in 11% of patients due to adverse reactions. The most common adverse effects included rash, dyspnea, fatigue, muscle and skeletal pain or edema [228]. Another humanized BsAb approved by the FDA in 2021 along the fast-track is zenocutuzumab (MCLA-128). The target of this BsAb is HER2 on another epitope than trastuzumab and HER3. This drug appears promising in the monotherapy in patients with gastric cancer, with progression following an earlier treatment [229]. It is furthermore characterized by good tolerance. This drug is also subject to testing in terms of combined therapy with hormonal therapy with trastuzumab and vinorelbine [230].

Since 2011, when the FDA approved ipilimumab, the first antibody targeting immune checkpoints, other antibodies currently used in the clinic have appeared, such as pembrolizumab, nivolumab, as well as durvalumab and atezolizumab [231] (Table 1). In the USA, they are used both as monotherapy and in combination therapy with conventional anticancer drugs for about 70 types of cancer [232]. Immunotherapy is usually well tolerated, the most common side effects are rash, fatigue or diarrhea. Importantly, immuno-oncology therapies provide documented clinical benefits in comparison with chemotherapy. They provide information on applications and clinical potential, helping clinicians understand the importance of newer therapeutic options. Immune checkpoint inhibitors used in the treatment of brain tumors may facilitate the presentation of tumor-associated antigens, resulting in an improved response in patients treated for brain glioma [233]. Consequently, the pre-operative approach used may enhance antigen presentation as well as allow the immune checkpoint inhibitor to penetrate the blood–brain and blood–tumor barrier, thereby increasing immune cell infiltration to further sensitize brain tumors and micrometastases to immunotherapies [234].

However, many patients still fail to respond to these effective antibody therapies due to primary or secondary resistance associated with the tumor cells, e.g., induction of alternative intracellular pathways, reduced expression of therapeutic target particles [231]. The phenomenon of resistance to treatment with antibodies may also be related to the cancer microenvironmental effect, as discussed in Section 7.

Almost all CAR T cell therapies used in the clinic are dedicated mainly to B-cell leukemia and target the CD19 antigen. Several therapies have been developed for this leukemia: tisagenlecleucel has been approved for the treatment of pediatric patients with refractory B-ALL [235], then for patients up to 25 years of age with relapsed B-ALL and for adults with diffuse large B-cell lymphoma [236]. Axicabtagene ciloleucel [237] has also been approved for adults with refractory diffuse large B cell lymphoma. In 2020, the FDA approved brexucabtagene autoleucel for patients with mantle cell lymphoma [238,239] while, in 2021, it approved B-cell maturation antigen (BCMA)-directed autologous CAR T cells (idecabtagene vicleucel) for patients with multiple myeloma [240]. Characteristic of this type of therapy are the high response rates, and, despite the fact that it may even cause serious side effects, as mentioned in chapter 4, it is of great interest among clinicians due to its high therapeutic potential.

The situation is different in the case of solid tumors, for which this therapy is ineffective. One problem is that CAR T cells can only recognize extracellular antigens. Improving the effects of therapy in solid tumors may be achieved after the development of CAR T cells targeting multiple therapeutic targets or by finding suitable neoantigens. Another obstacle in using this therapy on a large scale is its high cost, which makes the therapies unattainable for poorer societies.

Of the oncolytic viruses studied so far, one of the aforementioned T-VECs (IMLYGIC^®^, Amgen Inc., Southend Oaks, CA, USA) has been used, which has proved to be relatively effective in the treatment of melanoma as an alternative to other therapies. Clinical studies show that the use of oncolytic viruses together with other therapies may improve the prognosis of patients [241]. The activity of viruses consisting in causing the lysis of tumor-specific cells together with the stimulation of the immune system acts as a potential in situ anti-cancer vaccine. In the case of therapy with oncolytic viruses, the risks associated with the use of potential pathogenic particles should be taken into account, and despite the “devirulence” of oncolytic viruses, care should be taken when using them. Moreover, one type of oncolytic virus is not sufficient to destroy all cancer cells due to the heterogeneity of cancerous tissues and the complexity of cancer cells. Selected cancer cells and non-transformed support cells may be resistant to certain oncolytic viruses, indicating that one type of virotherapeutic agent may not be effective for all types of cancer. Limited identification of the virus and methods of its delivery to an individual patient vary [242].

## 11. Conclusions

Reaching the limits of the effectiveness of chemotherapy and its toxicity to normal tissues prompts the search for new treatment regimens based on personalized and combination therapy, which are the future of medicine. The evolution of cancer therapies is ensured by modern methods of molecular biology, enabling a better understanding of the biology of cancer and finding an appropriate therapeutic target, such as the NGS method. This progress is also possible thanks to the use of better and better bioinformatics methods, which enable the precise adjustment of the drug to the therapeutic target.

It can be assumed that biological therapy in the broad sense will play an increasingly important role in the treatment of neoplasms. In the review of selected studies, we present a number of methods for modifying CAR T cells therapy, anti-cancer vaccines, and antibody structure to improve their bioavailability, binding strength or stability. Moreover, attention was drawn to the directions of research that may contribute to the improvement of biological therapy effectiveness. In particular, the need to look at cancer in the context of the microenvironment, which is justified in the research results, was emphasized. The benefits of combining various biological therapies, e.g., immune checkpoint inhibitors with oncolytic viruses and anti-cancer vaccines, have been demonstrated.

## Figures and Tables

**Table 1:** Immune checkpoints and their inhibitors. CTLA-4—Cytotoxic T-Lymphocyte-associated Antigen 4, PD-1—Programmed Death 1, PD-L1—Programmed Death-Ligand 1.

Type of Immune Checkpoint	Stage of the Immune Response	Place of Action	Presence on Cells	Inhibitors of Immune Checkpoint
CTLA-4	Early activation phase	Lymph nodes	Activated T and B cells	Ipilimumab
PD-1	The effector phase	Periferal tissue, cancer imcroenvironment	T and B cells, natural killer cells, myeloid-derived suppressor cells	Nivolumab, Pembrolizumab,
PD-L1	The effector phase	Periferal tissue, cancer imcroenvironment	Antigen presenting cells, T and B cells, natural killer cells, myeloid-derived suppressor cells, hematopoietic cells, cancer cells	Atezolizumab, Durvalumab, Avelumab

**Table 2:** Cancer vaccines investigated in preclinical and clinical studies.

Type of Vaccine	Problems in Applicaion	Clinical Application	Origin
Dendritic cells	Huge cost	PROVENGE^®^, castration-resistant prostate cancer, approved by FDA	From patient’s PBMCs
Peptide/protein	Limited efficacy (intracellular processing)	No application	Synthetic peptides
DNA	Risk of insertional mutagenesis, limited efficacy	No application	From autologus tumor cells
mRNA	Instability (enzymatic degradation)	No application	From autologus tumor cells, tumor cRNA libraries

## Data Availability

Not applicable.
